# The relationship of Grasha–Riechmann Teaching Styles with teaching experience of National-Type Chinese Primary Schools Mathematics Teacher

**DOI:** 10.3389/fpsyg.2022.1028145

**Published:** 2022-10-20

**Authors:** Sze Hui Sim, Mohd Effendi Ewan Mohd Matore

**Affiliations:** ^1^Sekolah Jenis Kebangsaan Cina Kepong 2, Selangor, Malaysia; ^2^Faculty of Education, Research Centre of Education Leadership and Policy, Universiti Kebangsaan Malaysia (UKM), Bangi, Malaysia

**Keywords:** Grasha–Riechmann, teaching style, teaching experience, mathematics, teacher, relationship, National-Type Chinese Primary Schools

## Abstract

Grasha–Riechmann Teaching Styles have a high potential to be applied in Mathematics especially to help increase teacher educators’ knowledge. However, very little attention has been paid to the study of identifying the teaching style patterns of Mathematics teachers at the primary school National-Type Chinese Primary Schools or *Sekolah Jenis Kebangsaan Cina* SJKC. There is increasing concern about how this teaching style related to the teaching experience. This study aims to identify the patterns of Grasha–Riechmann Teaching Styles among primary school Mathematics teachers and the relationship between Grasha–Riechmann Teaching Styles with teaching experience. The quantitative approach through a survey was applied to 97 Mathematics teachers of SJKC Kepong, Kuala Lumpur using the simple random sampling method. The instrument was adapted from the Grasha–Riechmann Teaching Styles Questionnaire (1996), which measures five teaching styles such as Personal Model Teaching Style, Expert Teaching Style, Formal Authority Teaching Style, Delegator Teaching Style, and Facilitator Teaching Style. The patterns showed that the Personal Model Teaching Style is the most dominant, and the Facilitator Teaching as the least dominant style. The Spearman’s Rho Correlation also reported a very weak significant correlation between Grasha–Riechmann Teaching Styles with the teachers’ Mathematics teaching experience, specifically for Expert, Formal Authority, and Facilitator Teaching Styles. The study provides practical implications for educators’ professional development to diversify the training of teachers by experience and adapt them to the needs of student learning in primary school. These findings trigger ideas to get a better understanding by other demographic variables such as gender, age, and complexity of Mathematics subject.

## Introduction

Statistics of student enrolment in Science, Technology, Engineering, and Mathematics (STEM) in Malaysia reported an increasingly alarming downward pattern. Salhani (2019) found a decline in student involvement in STEM fields from a 49% increase in 2012 to a 44% drop in 2018. This scenario clearly shows a decline in the involvement of nearly 6,000 students a year in STEM fields. This indirectly contributes to the significant difference in student involvement with only 334,742 students in STEM fields in higher learning institutions compared to 570,858 students in non-STEM fields (Wan Faizal, 2019). The phenomenon of student decline in STEM fields is not in line with the target of the Malaysia Education Development Plan 2013–2025 to attract the interest and awareness of the community in STEM fields in the second wave of PPPM in 2016–2020. Although many initiatives have been undertaken by the ministry to increase the interest of students and the community in STEM fields, the pattern of decline remains.

Therefore, the role of Mathematics teachers in primary schools is very important in inculcating and nurturing students’ interest in STEM fields since the early schooling stage. Ainonmadiah et al. (2016) explained that teachers and students have close interpersonal relationships, where the teaching styles of teachers in the classroom are a component or element that ensures a sense of learning among students. In addition, the methods and practices of learning and facilitation (PdPc) are said to have a direct impact on students’ perceptions and concerns in mastering Science and Mathematics (Anis Humaira et al., 2019). This shows that the role of teachers in the presentation of PdPc to some extent influences students’ decisions and interests in the field of Mathematics.

Teacher quality gives the highest contribution to the success of a student. Based on a study by Nur Farhah and Fatimah Wati (2018), what students obtain is not dependent on the school attended but on the teachers at the school. Creative and innovative teaching practices can attract students (Rumainah and Faridah, 2017). Grasha (1996) also stated that the teaching styles of teachers are crucial for creating an ideal learning climate in the classroom. A good teaching style pattern among Mathematics teachers in primary schools is important so that the presentation of PdPc by the teachers can provide a positive perception and influence among students on the field of Mathematics. The literature also found a lack of research on the patterns of Grasha–Riechmann Teaching Styles among Mathematics teachers at the primary school level, particularly for National-Type Chinese Primary Schools (SJKC) compared to mainstream national schools.

Meanwhile, Normiati and Abdul (2019) opined that the teaching strategy of senior teachers is more traditional whereby the teaching experience factor that is honed by the interaction of the teachers’ work environment promises maturity and expertise, especially in the development of effective teaching. The analysis also found that teachers with 6 to 10 years of teaching experience complied with each instruction and implemented teaching in accordance with the guidelines of the education system. Meanwhile, the group of teachers with low experience, specifically 1 to 5 years of teaching experience, scarcely implemented such teaching and was not proficient in carrying out the teaching concept of 21st Century Learning (PAK21). Dewaele et al. (2018) also proved that teaching experience has a statistically significant influence on the creativity, classroom management, and pedagogical skills of teachers. More experienced teachers in the profession are also more creative in the classroom, are better at managing classroom activities, and report stronger pedagogical skills.

Nevertheless, there is a lack of empirical evidence on the link between teaching experience and the Grasha–Riechmann Teaching Styles among primary school Mathematics teachers, which has not been widely tested in the Malaysian educational context specifically for National-Type Chinese Primary Schools (SJKC). Most of the studies only explained in depth on teaching experiences that are just emphasizing on the certain group of teachers. However, it was very limited discussion on Mathematics primary school teacher. The lacking information will affect the effort on matching the teacher training quality with the teaching style in Chinese schools. In this case, the preference of teaching style by experience will help teachers to adapt creatively their teaching skill in Mathematics.

Accordingly, this study was conducted to investigate the relationship between Grasha–Riechmann Teaching Styles and the teachers’ Mathematics teaching experience.

Therefore, this study has two objectives:

- Determine the patterns of Grasha–Riechmann Teaching Styles practiced by primary school Mathematics teachers.
- Determine whether there is a significant relationship between Grasha–Riechmann Teaching Styles of Mathematics teachers with their Mathematics teaching experience.

Hence, the following hypotheses are constructed:

*Ho1:* There is no significant relationship between the overall Grasha–Riechmann Teaching Styles of Mathematics teachers with their Mathematics teaching experience.

*Ho2:* There is no significant relationship between the Expert Teaching Style of Mathematics teachers with their Mathematics teaching experience.

*Ho3:* There is no significant relationship between the Formal Authority Teaching Style of Mathematics teachers with their Mathematics teaching experience.

*Ho4:* There is no significant relationship between the Personal Model Teaching Style of Mathematics teachers with their Mathematics teaching experience.

*Ho5:* There is no significant relationship between the Facilitator Teaching Style of Mathematics teachers with their Mathematics teaching experience.

*Ho6:* There is no significant relationship between the Delegator Teaching Style of Mathematics teachers with their Mathematics teaching experience.

## Literature review

### Teaching styles

Mazaheri and Ayatollahi (2019) defined teaching styles as teachers’ preferred ways to solve problems, perform tasks, and make decisions in the teaching process. Heydarnejad et al. (2017) defined teaching style as teachers’ personal qualities and attitudes in teaching, which are reflected through the use of teaching techniques, activities, and approaches in teaching specific subjects in the classroom. In other words, the teaching style is a combination of motivation, personality, attitude, belief, and strategies in teaching (Karimnia and Mohammdi, 2019). Therefore, the teaching styles of teachers represent their behavior while teaching in the classroom and are one of the main determining factors for the success of student learning (Baradaran, 2016; Rosalia, 2017).

The teaching styles outlined by Grasha (1996) refer to the beliefs, behaviors, and needs of teachers that emerge in an educational context. Grasha believed that a teacher’s teaching style reflects the personal qualities of the teacher in terms of how to teach, guide, and direct the teaching process, thus impacting students and their ability to learn. In general, the success or failure of students is associated with a teacher’s teaching style, which is directly related to the teaching methods used during teaching. Indirectly, the teaching style turns into one of the components of a comprehensive transfer of teaching content. The teaching style may also be influenced by factors such as educational background, teaching experience, cultural background, and individual personal interests (Nouraey and Karimnia, 2016; Tavakoli and Karimnia, 2017). These factors can be identified by observing and studying teacher behavior.

Literature related to teaching styles displays various theories, models, and categorization of teaching styles through the use of different terminologies. For example, this includes the categorization of teaching styles to a didactic direct style and a student-centered indirect style (Flanders, 1970), Formal-Informal (Bennett et al., 1976), Open-Traditional (Solomon and Kendall, 1979), Intellectual Excitement-Interpersonal Rapport (Lowman, 1995), and Expert, Formal Authority, Personal Model, Facilitator, and Delegator Teaching Styles (Grasha, 1996) are among the few terminologies used to better explain these constructs. In the present study, only the Grasha–Riechmann Teaching Style model is discussed because it has potential in the context of Mathematics teaching.

### Grasha–Riechmann Teaching Styles (1996)

Grasha (Heydarnejad et al., 2017) argued that teaching styles involve constant teacher behavior in interaction with students during the teaching-learning process. Grasha described teaching styles as a criterion for personal qualities and behaviors that govern the way teachers manage classes. Hence, it can be said that teaching styles consist of all techniques, activities, and teaching approaches used by a teacher in the teaching process. The five dimensions of the Grasha–Riechmann Teaching Styles are Expert Teaching Style, Formal Authority Teaching Style, Personal Model Teaching Style, Facilitator Teaching Style, and Delegator Teaching Style. The dimensions or attributes of the Grasha–Riechmann Teaching Styles in terms of teacher roles, student characteristics, and the aspect of the advantages and disadvantages of each teaching style are explained in the following sub-sections.

#### Expert Teaching Style

Grasha (1996) argued that teachers with the Expert Teaching Style have knowledge and expertise on what students want to learn. The Expert Teaching Style makes teachers maintain their status as experts among their students by displaying accurate and comprehensive knowledge. In this regard, teachers with the Expert Teaching Style encourage students to face challenging situations to develop competencies in learning. As an expert, teachers also play a role in conveying information and expecting students to learn what they receive and take advantage of the information presented. Teachers are also careful in communicating information and will ensure that students are always ready.

In the Expert Teaching Style of Bergil and Erçevik (2019) can be seen as an advantage where teachers have accurate and comprehensive knowledge, skills, and information on the scope of targets to be taught to students. This comprehensive consolidation of knowledge, information, and skills can benefit experienced students. However, it should be emphasized that excessive use of Expert Teaching Styles may scare and curb the learning of students who are inexperienced or do not have a basic knowledge of the expected target topic. Furthermore, the presentation of knowledge or information conveyed by the teachers may not be of interest and motivation to the students at all. Additionally, the display of teacher knowledge and skills may not always show students the implicit thought process that produces an answer.

#### Formal Authority Teaching Style

Grasha (1996) reported that Formal Authority Teaching Style requires teachers to have status or position among students. This is because teachers are considered members of schools or faculties who contribute to the teaching and learning process by providing positive and negative feedback to the students. In this context, the teachers create concrete learning situations by setting learning objectives, rules, expectations, and principles of learning for students. Accordingly, teachers with the Formal Authority Teaching Style focus on preparing students with the necessary thinking structure in learning. The teachers also care about the correct, accepted, and standard way of doing things. Thus, students can be motivated through quality, effective, and meaningful learning methods.

The main advantages of the Formal Authority Teaching Style are emphasized on teacher expectations, methods, and standard ways to do things during the teaching and learning process. However, the use of the Formal Authority Teaching Style can also result in limited, permanent, and inflexible student engagement in the learning process. Hence, a strong attachment to the Formal Authority Teaching Style can contribute to rigid, standard, and less flexible ways of managing students.

#### Personal Model Teaching Style

Grasha (1996) explained that Personal Model Teaching Style refers to teachers who teach based on their own example. They will directly guide and encourage students to emulate it. Teachers with Personal Model Teaching Style set the prototypes for thinking and behaving. In this regard, the teachers constantly supervise, guide, and instruct students by showing them how to do things. In doing so, the teachers motivate students to observe, imitate, or reflect on the methods and approaches provided by them. The need for direct observation and imitation by students is the main strength of the Personal Model Teaching Style (Bergil and Erçevik, 2019). Teachers with Personal Model Teaching Style encourage students to observe and then imitate the teachers’ approach that is considered appropriate. However, some teachers may believe that their approach is the best and this consequently makes some students feel that they have low capacities if they cannot meet those expectations and standards. As a result, the students will feel less confident and demotivated in learning that exceeds their ability.

#### Facilitator Teaching Style

Grasha (1996) stated that the Facilitator Teaching Style emphasizes teacher and student interaction. Therefore, teachers with this teaching style act as facilitators in the classroom. They guide students by asking questions, exploring options, suggesting alternatives, and encouraging students to make informed decisions. The main goal of teaching is to nurture students who are independent and have high self-efficacy, where teachers encourage students to initiate and carry out their own responsibilities in learning. The choices, questions, and opportunities provided by the teachers serve as a guide and lead the students in learning situations. In the Facilitator Teaching Style, students can develop their own learning criteria. This style also shows that teachers are more likely to guide students to carry out project-based activities and provide optimal motivation to students. In this regard, the teachers work with the students on project assignments on a consultative basis by providing support and encouragement to the students. The main strength of the Facilitator Teaching Style is that the personal flexibility given by teachers is focused on the needs and objectives of student learning (Bergil and Erçevik, 2019). This will enable students to explore alternative options and methods of action. Nonetheless, the main drawback of the Facilitator Teaching Style is that it is time-consuming. Teachers and students may need more time, especially in the implementation of practical activities or project assignments. In addition, the Facilitator Teaching Style may also become ineffective when a more direct approach is needed. In fact, students may feel uncomfortable if this mechanism is not used positively and in a motivational manner.

#### Delegator Teaching Style

Delegator Teaching Style refers to teachers who emphasize the development of a student’s self-capacity. Students will be encouraged to conduct self-learning such as projects and teachers will act as a source of reference. The Delegator Teaching Style aims to develop students’ competencies by giving them autonomous characteristics. In this style, students are expected to work on projects independently and function as members with autonomous powers within their group. When the students need help, they can refer to teachers as a source of information to meet their needs (Grasha, 1996).

In the use of the Delegator Teaching Style, students consider themselves independent, capable, and autonomous. As a result, each student has the opportunity to become initiative and self-reflect by evaluating himself or herself. Nevertheless, teachers are sometimes confused about students’ willingness to take responsibility and face the need for autonomy. The students among youth also need to self-assess their ability to face adversity in life (Mohd Effendi Ewan et al., 2021). This situation can indirectly cause the students to feel worried and anxious in their efforts to carry out the tasks given by the teachers. Therefore, as a weakness, it should be borne in mind that students may not have the desired ability to fulfill their autonomous obligations. Besides, students may need rigorous supervision and intensive encouragement to overcome a sense of anxiety and reform themselves in learning norms.

## Present study

Studies on the patterns of Grasha–Riechmann Teaching Styles were found to be very limited both locally and internationally. Over the most recent 5 years, fewer research reports were found to identify the relationship of teaching experience with the Grasha–Riechmann Teaching Styles among teachers. In fact, international research on the Grasha–Riechmann Teaching Styles of teachers is barely focusing on teaching experience but is more likely to focus on teacher creativity and burnout (Ghanizadeh and Jahedizadeh, 2016), teacher self-efficacy (Baleghizadeh and Shakouri, 2017), student academic achievements (Khalid et al., 2017; Martin, 2019), student motivation (Massaada, 2016; Rosalia, 2017), as well as teachers’ behavior management and instructional management (Kazemi and Soleimani, 2016).

In Malaysia, one study had conducted to identify the Grasha–Riechmann Teaching Styles practiced among science lecturers of pre-university colleges in Penang (Anis Humaira et al., 2019). The findings found that the dominant teaching style of the Science lecturers was the Expert Teaching Style, followed by the Personal Model Teaching Style, Facilitator Teaching Style, and Delegator Teaching Style. Meanwhile, the Formal Authority Teaching Style was least practiced by lecturers. The Spearman’s Rho analysis found that it has no significant relationship between teaching styles and teaching experience (*r* = 0.089) with professional qualifications (*r* = 0.193). Ainonmadiah et al. (2016) also reviewed the relationship between Grasha–Riechmann Teaching Styles and the level of skipping secondary school in Bachok District, Kelantan. The findings showed that the most dominant category of teaching style practiced by the teachers was the Personal Model Teaching Style, followed by the Delegator Teaching Style, Expert Teaching Style, Formal Authority Teaching Style, and Facilitator Teaching Style. The analysis proved that there was a significant relationship related to the teaching styles of teachers based on the flow of subjects. However, Pearson’s Correlation Test showed no significant link between teaching styles and the level of skipping school (*r* = 0.062).

Another study by Mazeni and Hasmadi (2017) has identified the Grasha–Riechmann Teaching Styles practiced by Kemas Kindergarten teachers in Kelantan. A total of 50 kindergarten teachers were selected through strata sampling. The findings showed Delegator Teaching Style as the main teaching style, Facilitator as the second teaching style, followed by Personal Model, Expert Teaching Styles, and finally Formal Authority Teaching Style. The mean score achievement proved that the majority of the teachers used Delegator Teaching Style, while the least used teaching style was the Formal Authority Teaching Style. Teachers at Kemas Kindergarten used the Delegator Teaching Style because they had no formal educational background in early childhood education. In a different study, Nur Liyana and Zakiah (2017) findings also suggested that the Personal Model Teaching Style was most dominant.

As for the global context, Alami and Ivaturi (2016) in Oman reviewed a comparison of the Grasha–Riechmann Teaching Styles used by English lecturers at the Salalah College of Technology (SCT). The findings showed that the lecturers used different Grasha–Riechmann Teaching Styles, where the Expert Teaching Style was the dominant teaching style. It proved that the lecturers preferred to act as experts by displaying knowledge, disseminating information, and encouraging students to apply the information provided to them. Furthermore, this study found that lecturers with less than 5 years of teaching experience preferred to use the Facilitator Teaching Styles compared to other teaching styles. The Delegator Teaching Style was also used by lecturers with 5–10 years of teaching experience. However, overall, the Chi-Square Test proved no great connection between Grasha–Riechmann Teaching Styles and the teaching experience of English lecturers.

In Iran, Mazaheri and Ayatollahi (2019) conducted a study to explore the relationships among brain dominance, teaching experience, and the Grasha–Riechmann Teaching Styles of English teachers. This study involved 100 Iranian English teachers who taught at Shiraz high school. The results showed significant links among brain dominance, teaching experience, and the Grasha–Riechmann Teaching Styles of teachers. The results of Pearson’s correlation showed a significant moderate relationship between teaching experience and the Grasha–Riechmann Teaching Styles of teachers (*r* = 0.324, *p* < 0.05). It was also found that the teaching experience can predict the teaching style of a teacher with a beta value of 0.265. Meanwhile, in Turkey, Bergil and Erçevik (2019) concluded that the Personal Model and Facilitator Teaching Styles with 32.4% and 35.3%, respectively, were the choices of the English teachers. On the other hand, the Delegator and Expert Teaching Styles with 5.9% and 11.8%, respectively, were less applied by the teachers.

Another study in Spain (Fernandez-Rivas and Espada-Mateos, 2019) has analyzed the use of Grasha–Riechmann Teaching Styles among Physical Education teachers with reference to teaching experience and age of the teachers. The study used a sample of 455 Physical Education teachers covering a wide range of age groups and teaching experiences. The findings showed a significant relationship between the use of Grasha–Riechmann Teaching Styles among teachers and their teaching experience. The study also showed that teachers who have worked between 1 and 20 years used Facilitator and Delegator Teaching Styles more often than the teachers with more than 21 years of experience at school. In addition, the results proved that younger and less experienced teachers regularly used traditional teaching styles.

In addition, Martin (Martin, 2019) determined the dominant teaching style and the relationship between the teachers’ grade levels and their teaching styles. Based on the findings of the study, the Personal Model Teaching Style was the most dominant teaching style among respondents. The Expert and Formal Authority Teaching Styles were often used by Level Two primary school teachers, while the Personal Model and Facilitator Teaching Styles were applied by Level One primary school teachers. Furthermore, the Facilitator and Personal Model Teaching Styles were also seen to produce the highest academic achievement in Mathematics.

In another study, Massaada (2016) analyzed the Grasha–Riechmann Teaching Styles applied by English teachers and their influence on student motivation at Majene State High School 2. The results showed that English teachers in Majene State High School 2 applied four teaching styles, namely Expert Style, Formal Authority, Personal Model, and Facilitator. Meanwhile, the dominant teaching styles in this study were Expert, Personal Model, and Formal Authority Teaching Styles.

Rosalia (2017) conducted a study to identify the Grasha–Riechmann Teaching Styles applied by English teachers as well as the most influential teaching style toward the motivation of students at SMK Negeri 5 Makassar. The results showed that English teachers at SMK Negeri 5 Makassar applied three teaching styles, namely Expert Teaching Style, Formal Authority Teaching Style, and Facilitator Teaching Style. The researchers found that the most influential teaching style toward student motivation was the Facilitator Teaching Style, which focuses on teacher-student interaction. The results also showed that the teachers tried to change their teaching style by applying fun activities.

Previous research on Grasha–Riechmann Teaching Styles has also been conducted by Kazemi and Soleimani (2016). The researchers randomly selected 103 English teachers working in private language learning centers. The results showed that Iranian English teachers followed the intervention class management approach and most of them used the Formal Authority Teaching Style. The Grasha–Riechmann Teaching Styles of teachers were also found to correlate significantly with their behavior management and instructor management.

Based on the study in Malaysia and worldwide, it can be concluded that there were clear gaps in past studies on the exploration of the Grasha–Riechmann teaching style patterns among primary school Mathematics teachers to be filled on the context of SJKC. Hence, this study is relevant to be carried out to increase the empirical evidence of existing knowledge in the teaching styles for Chinese-based type of school.

## Materials and methods

The study used a survey with quantitative approach through questionnaires. Questionnaires were given to obtain respondents’ feedback on the Grasha–Riechmann Teaching Styles among primary school Mathematics teachers. In this study, the independent variables include the teaching experience, while the dependent variable is the Grasha–Riechmann Teaching Styles of Mathematics teachers.

### Samples

The population for this study involves Mathematics teachers from National-Type Chinese Primary Schools (SJKC) Kepong 1, Kepong 2, and Kepong 3 Sentul Zone, Kuala Lumpur. The total population of Mathematics teachers from three primary schools in Kepong is 130. Based on the sample calculation *via* Raosoft version 3.1.9.4 statistical software, the alpha value was recorded at 0.05. The calculated results further estimated a minimum sample size of 130 respondents; however, after considering a 50% possible loss rate (respondents refusing to participate or withdrawing), the recommended sample size for this study entails 97–98 respondents. In this study, random sampling was used easily for selecting the respondents of the study as it involves the selection of respondents with important knowledge or information that is consistent with the purpose of the study (Donkor, 2019). In the context of this study, the selected teachers only involve Mathematics teachers at the SJKC level in Kepong, Kuala Lumpur.

### Instrumentation

The study used an instrument adapted from the Grasha–Riechmann Teaching Styles Questionnaire (1996; Grasha, 1996). Donkor (Donkor, 2019) explained that the use of questionnaires is appropriate for obtaining data and information quickly, especially when a large number of respondents is required. The instrument outlines five constructs that comprise five different types of teaching styles (Expert Teaching Style, Formal Authority Teaching Style, Personal Model Teaching Style, Facilitator Teaching Style, and Delegator Teaching Style). The instrument with a seven-point Likert scale was modified to a five-point Likert scale to avoid confusion and facilitate the interpretation or preference of the respondents. In addition, the researchers translated the questionnaire from English to Bahasa Malaysia through a direct translation. The instrument has also undergone face validity and content validity processes by experts in Pedagogy and Mathematics. Besides, the reliability aspect of the instrument was tested using Cronbach’s Alpha analysis to test for internal consistency. Mailizar (2018) stated that internal consistency refers to consistency in instruments. All the 40 items in instrument had good internal consistency (Cronbach’s Alpha from 0.64 to 0.83) and high reliability (coefficient from 0.65 to 0.86). The respondents’ demographics such as gender and teaching experience are included in the questionnaire. Overall, the instrument comprises 40 items, specifically eight items in each construct. The distribution of the items by construct is depicted in Table 1.

**Table 1:** Distribution of items by construct.

Teaching style	Item number	Number of items
Expert Teaching Style	1–8	8
Formal Authority Teaching Style	9–16	8
Personal Model Teaching Style	17–24	8
Facilitator Teaching Style	25–32	8
Delegator Teaching Style	33–40	8
Total		40

The questionnaire consists of two sections, namely Section A (teacher information) and Section B (Grasha–Riechmann Teaching Styles of teachers). The score for each item is determined by the respondents’ responses on a 5-point Likert scale: 1 = Strongly Disagree; 2 = Disagree; 3 = Neither Agree nor Disagree; 4 = Agree; and 5 = Strongly Agree. The questionnaire used the Likert scale because this scale is easy to administer and takes a while to receive feedback from the respondents (Seng et al., 2016).

### Administration

For the administration of data collection, permission and approval were officially obtained from the Department of Education Office (JPP) Sentul Division, Kuala Lumpur. Subsequently, a pilot study was carried out to test the validity and reliability of each item in the questionnaire. A total of 35 Mathematics teachers from the SJKC in Keramat Zone, who have the same profile characteristics as the actual respondents, were selected as respondents in the pilot study. The Grasha–Riechmann Teaching Styles Questionnaire was prepared in Google Forms and the questionnaire link was given to the mathematics teachers involved. On average, the pilot study respondents completed the questionnaire within 10 to 15 min. Through this pilot study, flaws and confusion found in the questionnaire such as spelling errors, the ambiguity of the meaning of statements, and fewer clear instructions were improved based on the comments given. The respondents indicated that the items presented in the questionnaire were easy to understand and this proves that the face validity has been met. Subsequently, content validity through experts and the actual study were conducted.

## Data analysis

### Data preparation

The quantitative data were processed using IBM Statistical Package for Social Science (SPSS) version 26.0 software. Descriptive analysis and inference analysis were used to obtain the results. Data analysis based on the research question is shown in Table 2.

**Table 2:** Research objectives and data analysis.

Research objective	Data analysis
Determine the pattern of Grasha–Riechmann Teaching Styles practiced by primary school Mathematics teachers.	Frequency and descriptive
Determine whether there is a significant relationship between the Grasha–Riechmann Teaching Styles of Mathematics teachers with their Mathematics teaching experience.	Spearman rank

For the first objective, descriptive frequencies and mean scores of the five constructs of Grasha–Riechmann Teaching Styles were obtained and compared to identify the patterns of Grasha–Riechmann Teaching Styles practiced by the respondents. For the second objectives, some considerations were made prior to the use of parametric tests such as Pearson’s Correlation. One of the main considerations is the normality of data. In the context of this study, non-parametric tests such as Spearman Rank were used because the Kolmogorov–Smirnov Test showed that the data did not meet the normal distribution requirement due to a significance level of *p* = 0.000 (*p* < 0.05). Accordingly, the Spearman Rank Tests were selected as an alternative to parametric testing.

The correlational strength was determined based on Chua’s (Chua, 2021) interpretation. According to Chua (2021), correlation coefficient (r) refers to the measurement value of the relationship between two variables and this r value ranges between +1.00 and −1.00. Since perfect correlation is rare in research, correlation coefficients are reported in two decimal points. Table 3 shows the strength levels of correlation coefficient values (r).

**Table 3:** Strength level of correlation coefficient size.

Correlation coefficient size (r)	Correlation strength
0.91 to 1.00 or −0.91 to −1.00	Very strong
0.71 to 0.90 or −0.71 or −0.90	Strong
0.51 to 0.70 or −0.51 to −0.70	Moderate
0.31 to 0.50 or −0.31 to −0.50	Weak
0.01 to 0.30 or −0.01 to −0.30	Very weak
0.00	No correlation

## Results

The profile of the respondents is shown in Table 4. The number of female respondents is 42 (43.3%), while the number of male respondents is 55 (56.7%). Most of the respondents were 21–30 years old, which comprise 40 respondents with 41.2%. Meanwhile, respondents aged 31–40 years and 51–60 years constituted 23.8% and 20.6%, respectively, and those aged 41–50 years only constituted 14 respondents with 14.4%. As for the respondents’ Mathematics teaching experience, a total of 53 respondents (54.6%) had 1–10 years of teaching experience in the mathematics subjects. This is followed by 18 respondents (18.6%) with 11–20 years of teaching experience and 15 respondents (15.5%) with 21–30 years of Mathematics teaching experience. Meanwhile, only 11 respondents (11.3%) had more than 31 years of experience in teaching Mathematics subjects.

**Table 4:** Demographic profile.

Demographic profile	*N*	%
**Gender**		
Female	42	43.3
Male	55	56.7
**Age**		
21–30 years old	40	41.2
31–40 years old	23	23.8
41–50 years old	14	14.4
51–60 years old	20	20.6
**Mathematics teaching experience**		
1–10 years	53	54.6
11–20 years	18	18.6
21–30 years	15	15.5
31 years and above	11	11.3
Total	97	100

For the first objective, the scores of frequencies, mode, mean average, and standard deviation for the five constructs of the Grasha–Riechmann Teaching Styles of teachers were obtained and compared to identify the patterns of the Grasha–Riechmann Teaching Styles practiced by primary school Mathematics teachers. The respondents’ responses to the five Grasha–Riechmann Teaching Style constructs were explained separately. Table 5 shows that the majority of respondents agreed and strongly agreed with almost all items, alongside the mode value of 4 representing the scale of an agreement to all items, except Item 7 for the Expert Teaching Style construct. The item “I give students negative feedback if the performance is unsatisfactory” recorded a mode value of 2, which suggests that the majority of the respondents were not in agreement with this statement. The mean scores also indicated that the dominant teaching style adopted by the primary school Mathematics teachers is Personal Model Teaching Style (Mean = 4.12, Standard Deviation = 0.62), followed by the Expert Teaching Style (Mean = 3.87, Standard Deviation = 0.69), Formal Authority Teaching Style (Mean = 3.86, Standard Deviation = 0.65), Delegator Teaching Style (Mean = 3.79, Standard Deviation = 0.69), and finally the Facilitator Teaching Style (Mean = 3.77, Standard Deviation = 0.76). This proves that the mathematics teachers were more inclined to the Personal Model Teaching Style than other teaching styles. However, these teachers used the Facilitator Teaching Style the least.

**Table 5:** Frequency and descriptive analysis for Grasha–Riechmann Teaching Styles.

	Item code	Strongly disagree	Disagree		Neither agree nor disagree		Agree		Strongly agree			Mode	Mean	SD
		f	%	f	%	f	%	f	%	f	%			
Expert Teaching Style	S1	0	0	6	6.2	3	3.1	51	52.6	37	38.1	4	3.87	0.69
	S2	0	0	3	3.1	32	33	46	47.4	16	16.5	4		
	S3	0	0	0	0	12	12.4	67	69.1	18	18.6	4		
	S4	0	0	6	6.2	5	5.2	72	72.4	14	14.4	4		
	S5	0	0	1	1	16	16.5	76	78.4	4	4.1	4		
	S6	0	0	0	0	9	9.3	55	56.7	33	34.0	4		
	S7	12	12.4	35	36.1	23	23.7	18	18.6	9	9.3	2		
	S8	0	0	0	0	7	7.2	77	79.4	13	13.4	4		
Formal Authority Teaching Style	M1	0	0	6	6.2	16	16.5	65	67	10	10.3	4	3.86	0.65
	M2	0	0	0	0	9	9.3	74	76.3	14	14.4	4		
	M3	0	0	0	0	17	17.5	70	72.2	10	10.3	4		
	M4	0	0	3	3.1	15	15.5	71	73.2	8	8.2	4		
	M5	0	0	1	1.0	7	7.2	79	81.4	10	10.3	4		
	M6	0	0	6	6.2	13	13.4	65	67.0	13	13.4	4		
	M7	6	6.2	7	7.2	16	16.5	55	56.7	13	13.4	4		
	M8	0	0	1	1.0	37	38.1	47	48.5	12	12.4	4		
Personal Model Teaching Style	S1	0	0	8	8.2	24	24.7	45	46.4	20	20.6	4	4.12	0.62
	S2	0	0	1	1.0	10	10.3	59	60.8	27	27.8	4		
	S3	0	0	0	0	6	6.2	65	67.0	26	26.8	4		
	S4	0	0	0	0	7	7.2	54	55.7	36	37.1	4		
	S5	0	0	10	10.3	15	15.5	54	55.7	18	18.6	4		
	S6	0	0	0	0	1	1.0	74	76.3	22	22.7	4		
	S7	0	0	0	0	4	4.1	64	66.0	29	29.9	4		
	S8	0	0	0	0	3	3.1	73	75.3	21	21.6	4		
Facilitator Teaching Style	M1	6	6.2	11	11.3	19	19.6	42	43.3	19	19.6	4	3.77	0.76
	M2	6	6.2	0	0	11	11.3	73	75.3	7	7.2	4		
	M3	0	0	0	0	19	19.6	69	71.1	9	9.3	4		
	M4	0	0	7	7.2	9	9.3	71	73.2	10	10.3	4		
	M5	0	0	14	14.4	7	7.2	71	73.2	5	5.2	4		
	M6	0	0	31	32.0	13	13.4	43	44.3	10	10.3	4		
	M7	0	0	1	1.0	18	18.6	71	73.2	7	7.2	4		
	M8	0	0	0	0	11	11.3	65	67.0	21	21.6	4		
Delegator Teaching Style	S1	0	0	6	6.2	31	32.0	47	48.5	13	13.4	4	3.79	0.67
	S2	0	0	18	18.6	19	19.6	53	54.6	7	7.2	4		
	S3	0	0	7	7.2	36	37.1	48	49.5	6	6.2	4		
	S4	0	0	2	2.1	37	38.1	51	52.6	7	7.2	4		
	S5	0	0	0	0	19	19.6	59	60.8	19	19.6	4		
	S6	0	0	0	0	22	22.7	59	60.8	16	16.5	4		
	S7	0	0	0	0	17	17.5	59	60.8	21	21.6	4		
	S8	0	0	1	1.0	16	16.5	69	71.1	11	11.3	4		

To answer the second research question, Table 6 indicates that H_O1_, H_O2_, H_O3_, H_O4,_ H_O5,_ and H_O6_ have been rejected. The Spearman Rank test showed a very weak and significant positive correlation between the overall Grasha–Riechmann Teaching Styles and Mathematics teaching experience [r_s_(95) = 0.274, *p* = 0.007]. This also indicates the likelihood that the more teaching experience that the teachers have in mathematics, the higher application of Grasha–Riechmann Teaching Styles among them.

**Table 6:** Spearman’s rho correlation for Grasha–Riechmann Teaching Styles.

Grasha–Riechmann teaching style	Mathematics teaching experience		Decision
	Correlation coefficient	Sig. (2-tailed)	
Overall	0.274**	0.007**	H_1_ rejected
Expert Teaching Style	0.266**	0.009**	H_2_ rejected
Formal Authority Teaching Style	0.217*	0.033**	H_3_ rejected
Personal Model Teaching Style	0.062	0.548	H_4_ failed to reject
Facilitator Teaching Style	0.345**	0.001**	H_5_ rejected
Delegator Teaching Style	0.192	0.060	H_6_ failed to reject

** Correlation is significant at the 0.01 level (2-tailed).

**Figure 1:**
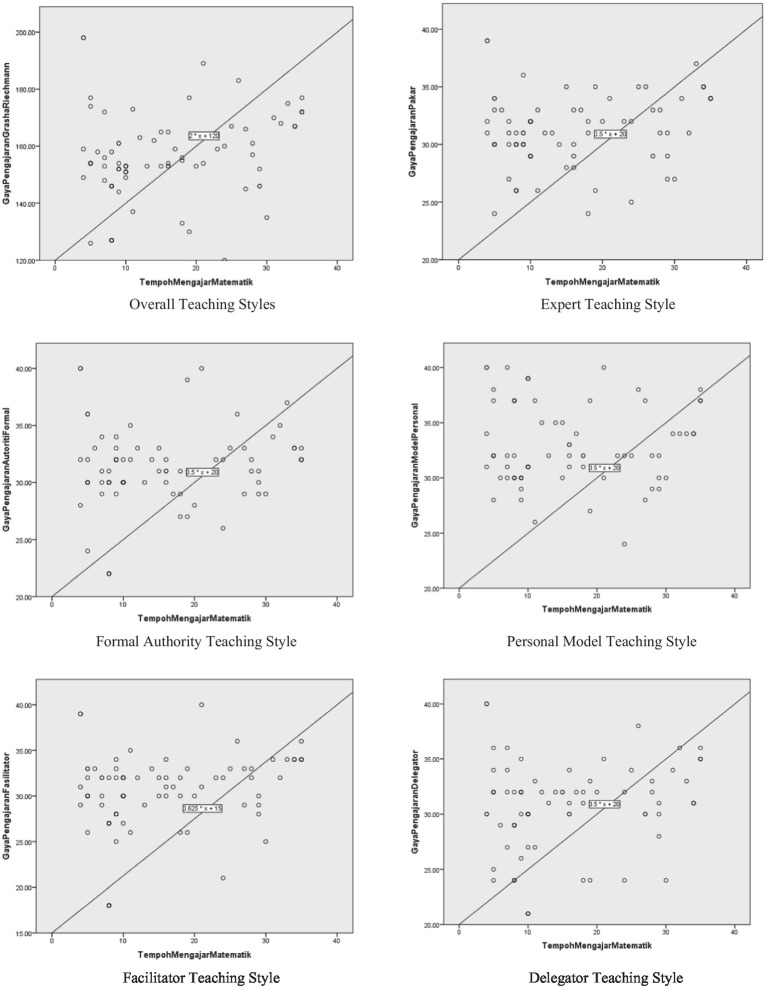
Scatter plots for the correlation between Mathematics teaching experience and Grasha–Riechmann teaching styles.

Looking at each construct of teaching style, interesting findings that can be noted are the significant positive relationships for the Expert Teaching Style [r_s_ (95) = 0.266, *p* = 0.009], Formal Authority Teaching Style [r_s_ (95) = 0.217, *p* = 0.033], and Facilitator Teaching Style [r_s_ (95) = 0.345, *p* = 0.001]. The highest correlation was recorded by the Facilitator Teaching Style, followed by the Expert Teaching Style and the Formal Authority Teaching Style. The two more hypotheses that failed to be rejected were the Personal Model Teaching Style and the Delegator Teaching Style. Thus, there was no relationship between the Grasha–Riechmann Teaching Styles for both constructs and Mathematics teaching experience, as reported in Table 6. Figure 1 shows the scatter plots for the correlation between Mathematics teaching experience and the Grasha–Riechmann Teaching Styles. Scattered data plots with fit lines indicated poor relationships of the variables involved.

## Discussion

This study focuses on identifying the patterns of Grasha–Riechmann Teaching Styles of primary school Mathematics teachers and the relationship between Grasha–Riechmann Teaching Styles with the teachers’ Mathematics teaching experience.

The findings for the first research objective have shown that the Personal Model Teaching Style was most practiced by primary school Mathematics teachers. On the other hand, the Facilitator Teaching Style was practiced the least. Literature on the patterns of the Grasha–Riechmann Teaching Styles among teachers is very limited both locally and abroad. The findings are consistent with past findings in which the Personal Model Teaching Style precedes other teaching styles and is the top choice for educators (Abdull Sukor et al., 2014; Ainonmadiah et al., 2016; Beers, 2016; Martin, 2019). Studies by Nur Liyana and Zakiah (2017) and Ghanizadeh and Jahedizadeh (2016) also reported similar findings by which the Personal Model Teaching Style is dominant compared to other teaching styles. The contribution of this study is seen to reinforce the findings on the Grasha–Riechmann Teaching Style patterns practiced by primary school SJKC teachers in Malaysia.

The findings clearly demonstrated the trend of using the Personal Model Teaching Style where the primary school Mathematics teachers would teach based on their own examples, such as setting a prototype for students to think and behave by giving direct guidance and subsequently encouraging the students to emulate them (Grasha, 1996). This style also encourages the environment to be student-centered teaching and learning process that involves an inquiry process in problem-solving questions that can increase mathematics performance (Suik Fern and Mohd Effendi Ewan, 2022). In addition, teachers with a high Personal Model Teaching Style were found to be very concerned with the students’ mastery of the lesson contents. They often demonstrated how to master the contents of lessons, concepts, and principles and relate them by giving examples based on their own experiences. In the context of Mathematics, the teachers preferred this teaching style as they can supervise, guide, direct, and motivate students to make observations and subsequently emulate the approach that the teachers have shown.

From a theoretical point of view, one of the logical explanations for the trend of using Personal Model Teaching Style among primary school Mathematics teachers in this study can be explained through Bandura’s Social Learning Theory (Bandura, 1962), which explains that humans naturally learn through the process of observation and imitation. This theory emphasizes the need for direct observation and imitation by students in the use of the Personal Model Teaching Style. In this study, the mathematics subjects require individual attention and regular training to help improve students’ academic achievements (Beers, 2016). Due to the conceptualized nature of Mathematics, primary school students who are in the early stages of learning are encouraged to observe and refer to the work steps or procedures shown by the teachers. The information they obtained is gradually stored in memory and released when they responded to the mathematical questions given to them.

Preliminary research on Grasha (1994) also found that the Personal Model Teaching Style was used dominantly by all instructors at all levels of academic education, be it professors, associate professors, tutors, or teachers. Besides, the Personal Model Teaching Style was found to have been dominantly used in teaching courses at all higher education levels compared to other teaching styles. This clearly shows that teachers tend to use the Personal Model Teaching Style characterized by a hands-on approach and encourage students to observe and replicate it (Abdull Sukor et al., 2014; Martin, 2019). In short, the Personal Model Teaching Style emphasizes teaching through observation and guidance where the teacher serves as a real model in controlled learning activities.

More interestingly, the frequent use of Personal Model Teaching Style among Mathematics teachers is also likely due to the potential of this teaching style toward the academic growth of students in Mathematics. Generally, the teaching style of teachers has a great impact on the motivation and achievement of students in a particular subject (Heydarnejad et al., 2017). To explain the relationship between Grasha–Riechmann Teaching Styles and the academic growth of students in Mathematics, Martin (2019) conducted a comprehensive quantitative correlation study on students’ academic achievements in 37 U.S. international schools. This study also showed that the Personal Model Teaching Style produced the highest growth in academic achievement in Mathematics. Meanwhile, in Malaysia, Abdull Sukor et al. (2014) conducted a study to identify the relationship between the teaching styles of lecturers and university students’ academic involvement. The study found that most students were more likely to engage in learning when the lecturers used the Personal Model Teaching Style to deliver their teaching.

The findings also showed the Facilitator Teaching Style as the least dominant teaching style among respondents. The findings are in line with Ainonmadiah et al. (2016) findings on teachers from five schools in Bachok District, Kelantan. The findings of this study can be explained through Piaget’s Theory of Cognitive Development (Piaget, 1936), which classifies the cognitive development of children at the age of seven to 12 years old as a level of concrete operation marked by the use of clear and logical rules. Children of this age are seen applying logical thinking over concrete objects, but neither in the abstract nor hypotheses. Hence, the children’s way of thinking is still limited as the focus is more on concrete objects and can only solve problems encountered directly (Astuti, 2018).

Therefore, the limited cognitive development of children at the primary school level has limited the use of the Facilitator Teaching Style that emphasizes students’ self-learning through the implementation of practical activities or project assignments. This is because children at this stage are less capable of solving hypothetical problems and abstract tasks (Lutz et al., 2018). If the Facilitator Teaching Style is applied by teachers regardless of the needs of the students’ level of learning, especially in primary schools, then it is very likely that this will result in the negative view of students of Mathematics due to the “Mathematical stress” factor experienced. Furthermore, the implementation of the teaching process to the physically and mentally unprepared group of students may also cause difficulties and waste of time. Hence, these factors are likely to contribute to the infrequent use of Facilitator Teaching Style among primary school Mathematics teachers. However, this explanation requires further research to be done in the future in order to empirically make stronger confirmation.

The current study also showed the implicit finding that primary school Mathematics teachers are more likely to apply teacher-centered teaching orientation (Personal Model Teaching Style, Expert Teaching Style, and Formal Authority Teaching Style) compared to student-centered teaching orientation (Delegator Teaching Style and Facilitator Teaching Style). However, this scenario is inconsistent with the demands of 21st Century Learning (PAK21) where both teacher-centered and student-centered teaching orientations should be balanced to achieve maximum student learning outcomes. The previous study also shows that the level of readiness of trainee teachers is high in terms of interest, knowledge, and skills in integrating PAK-21 in teaching and learning mathematics (Mazlini et al., 2021).

In a teacher-centered classroom, students become passive without having control over self-learning. In this context, teachers make all decisions regarding the curriculum, teaching methods, and assessments, thus hindering the development of student competencies and learning achievements (Dole et al., 2016; Goff, 2016; Lak et al., 2017). On the other hand, in a student-centered classroom, students are given more attention and responsibility for self-learning (Upadhya and Lynch, 2019). Student-centered strategies include techniques such as active learning, problem-solving through critical and creative thinking, role play, and group learning such as cooperative learning. Through this teaching orientation, students are given the opportunity to build deep knowledge and understanding of the learning contents, thus inculcating a positive attitude in the learning process (Alam, 2016). Hence, it is evident that student-centered teaching styles should not be ignored but require serious attention and the proactive steps of teachers to balance them with student learning, especially in today’s era of rapid and challenging development of educational transformation.

The findings for the second research objective showed a very weak and significant positive correlation between the Grasha–Riechmann Teaching Styles of Mathematics teachers with their Mathematics experience teaching. These findings confirm the relationship between the two variables presented in the conceptual framework of the study.

Based on the context of the five constructs of the Grasha–Riechmann Teaching Styles, the findings showed significant relationships among the Expert Teaching Style, Formal Authority Teaching Style, and Facilitator Teaching Style of teachers with their Mathematics teaching experience. However, there was no significant relationship involving the Personal Model Teaching Style and Delegator Teaching Style with the teachers’ Mathematics teaching experience. The findings directly provide an interpretation that the Grasha–Riechmann Teaching Styles of Mathematics teachers have a significant relationship with their Mathematics teaching experience, specifically for the Expert Teaching Style, Formal Authority Teaching Style, and Facilitator Teaching Style.

The findings of this study are consistent with past studies that examined the relationship between the Grasha–Riechmann Teaching Styles of teachers and their teaching experience. More interestingly, the context of this study that involves Mathematics teachers has similar results to past studies involving teachers in different courses, which have shown a significant relationship between the Grasha–Riechmann Teaching Styles of English teachers in public and private sectors in Iran with their teaching experience (Piaget, 1936; Hosseini Fatemi and Raoufi, 2014; Mazaheri and Ayatollahi, 2019). In different courses, a study by Kothari and Pingle (2015) on administrative instructors from various schools throughout India as well as a study by Sabado and Allan (2019) on Technical Vocational Education (TVE) teachers in the Philippines also found similar findings to the current study, in which the Grasha–Riechmann Teaching Styles of teachers had a significant relationship with their teaching experience. This proves that the Grasha–Riechmann Teaching Styles do not only have a significant relationship with the teaching experience of Mathematics teachers but also those specializing in other areas.

Theoretically, teachers’ teaching experience indeed had a statistically significant influence on the creativity, classroom management, and pedagogical skills of the teachers (Dewaele et al., 2018). More experienced teachers in the profession are more creative in the classroom, more skilled at managing classroom activities, and have stronger pedagogical skills. Hosseini Fatemi and Raoufi (2014) confirmed significant differences in the teaching styles between less experienced teachers (1–10 years of teaching experience) and more experienced teachers (over 15 years of teaching experience), where more experienced teachers had higher mean scores through the application of the Expert Teaching Style, Formal Authority Teaching Style, and Facilitator Teaching Style. More experienced teachers were also found to have a higher level of knowledge, expertise, and mastery of course materials in order to be able to act as experts who display comprehensive knowledge and deliver information effectively (Sabado and Allan, 2019).

In other words, more experienced teachers have accurate and comprehensive knowledge, skills, and information on the target scope to be taught to students, in line with the requirements to apply the Expert Teaching Style. Hence, this phenomenon is seen as a contributor that may lead to a significant positive correlation between the teaching styles of Mathematics teachers and their Mathematics teaching experience.

Based on a study by Bruno et al. (2019) that used administrative data for 10 years in Los Angeles, teachers with low teaching experience tended to face problems, especially in the aspect of teacher pedagogical skills. Compared to experienced teachers, inexperienced teachers faced difficulties in classroom management and in controlling student behavior during the Learning and Facilitation (PdPc) process. On the other hand, experienced teachers were more likely to engage in standard classroom management efforts and were able to create concrete learning situations by setting learning objectives for students (Faruji, 2012). Besides, more experienced teachers were also more concerned with accepted, accurate, and standardized ways of doing things as prioritized in the Formal Authority Teaching Style.

Past research has also shown that experienced teachers have a better understanding of student needs and are able to explore options to meet these needs of students (Sabado and Allan, 2019). Evidently, a deep understanding of the various needs of students helps experienced teachers build close interpersonal relationships with students (Rahimi and Asadollahi, 2021). Indirectly, experienced teachers can interact well with their students as well as guide and direct the students by asking questions, exploring options, and suggesting alternatives, in line with the characteristics of the Facilitator Teaching Style outlined by Grasha (2002). Accordingly, experienced teachers can become good facilitators compared to inexperienced teachers.

This could explain the findings in the context of this study, which illustrates a significant positive correlation relationship between the Facilitator Teaching Style of Mathematics teachers and their Mathematics teaching experience. However, a gap exists when the relationship between teachers’ teaching experience and the Grasha–Riechmann Teaching Styles of primary school Mathematics teachers has not been widely tested, regardless of the Malaysian educational context or abroad. Therefore, the findings require further studies in the future for a more robust confirmation through empirical evidence.

## Implication

The findings of this study provide theoretical implications by strengthening and expanding the concept of the Grasha–Riechmann Teaching Styles by proving Personal Model Teaching Style as the dominant teaching style compared to other teaching styles in the context of primary school Mathematics teachers, which is in line with Grasha (1996) initial study. In addition, implications for the body of knowledge also exist through new findings in terms of the differences in Grasha–Riechmann Teaching Styles based on gender as well as the relationship between teaching styles and teaching experience in the context of primary school Mathematics teachers. The added value is clearly obtained through a specific analysis of each Grasha–Riechmann Teaching Style. These findings have indirectly added value to existing knowledge and a deeper understanding of the Grasha–Riechmann Teaching Styles practiced by teachers.

Practical implications also exist as the findings of this study provide early information to educators in diversifying teaching styles and improving the learning needs of different students in the classroom. In facing the challenges of Industrial Revolution 4.0, the rapid transformation of education has demanded teacher initiation in transforming the traditional teacher-centric teaching method into a more student-centric teaching approach. In student-centered teaching orientation, students are given more attention and responsibility for self-learning. The main responsibility of a teacher is to build and maintain a conducive learning environment where students are encouraged to build their own knowledge while the teacher acts as a facilitator and guide. In this regard, teachers are encouraged to adopt student-centered strategies that include techniques such as active learning, problem-solving by engaging in critical and creative thinking, role play, and group or cooperative learning. Indirectly, students are able to build meaningful relationships among existing knowledge, new knowledge, and the processes involved in learning, which are in line with the demands of 21st Century Learning (PAK21).

Likewise, implications exist in the pedagogical practices of different teachers. In the context of this study, the Ministry of Education can enhance the pedagogical skills of novice teachers by formulating a more comprehensive, appropriate, and practical strategy to improve professionalism among teachers in Malaysia. Seminars on knowledge, skills, and professional practices such as Content and Methods of Pedagogical Subjects, Creativity, and Pedagogical Innovation as well as Instructional Leadership courses can be implemented to improve the quality of teaching styles among teachers.

## Conclusion

This study has revealed that primary school Mathematics teachers preferred the Personal Model Teaching Style, whereas the Facilitator Teaching Style was less popular among primary school Mathematics teachers. The findings also showed a very weak and significant positive correlation between Grasha–Riechmann Teaching Styles and teaching experience, which encompass the Expert Teaching Style, Formal Authority Teaching Style, and Facilitator Teaching Style. Besides, based on the results of this study, some things need to be taken seriously because the current study only covers a factor as the independent variable, namely teaching experience, which fall into the demographic category. However, past studies have highlighted that the Grasha–Riechmann Teaching Styles of teachers are not influenced by one aspect or factor alone. Therefore, more in-depth studies are needed because there is a possibility of other influential factors that have yet to be explored.

In this regard, further studies can be developed by examining other demographic factors such as school type, age, subject flow, professional qualifications, and socioeconomic status of teachers. The Grasha–Riechmann Teaching Styles can also be attributed to other factors such as emotion, self-efficacy, personality traits, creativity, attitudes, thinking style, and autonomy of teachers as well as student-related factors such as the level of reasoning, learning style, academic involvement, student interest or motivation, and the number of students in the classroom. Besides, the study also has delimitations that can be improved in the future. As the respondents in this study only involve Mathematics teachers from SJKC Kepong 1, Kepong 2, and Kepong 3 Sentul Zone, Kuala Lumpur. For future research, it was suggested to use a larger sample involving more types of schools. It may also consider a comparison between rural and urban schools as well as between regular and high-performance schools to look at the differences in Grasha–Riechmann Teaching Styles. A wider and larger population can provide a more comprehensive and detailed picture of the Grasha–Riechmann Teaching Styles of teachers. This study is also limited to primary school Mathematics teachers only; hence, future studies on the Grasha–Riechmann Teaching Styles should involve various subjects and other levels of study. In terms of methodology, the data were based on questionnaires only; thus, it is better to combine both qualitative and quantitative methods to obtain more comprehensive and meaningful findings to improve the teaching styles of teachers.

## Data availability statement

The original contributions presented in the study are included in the article/supplementary material, further inquiries can be directed to the corresponding author.

## Ethics statement

The studies involving human participants were reviewed and approved by Education Planning and Research Division, Ministry of Education Malaysia and Faculty of Education, UKM. Written informed consent for participation was not required for this study in accordance with the national legislation and the institutional requirements.

## Author contributions

SS and MM: conceptualization, validation, resources, and data curation. SS: methodology, software, formal analysis, investigation, writing—original draft preparation, and project administration. MM: writing—review and editing, visualization, supervision, and funding acquisition. All authors contributed to the article and approved the submitted version.

## Funding

This research was funded by the Faculty of Education, Universiti Kebangsaan Malaysia (UKM) through the grant of Ganjaran Penyelidikan (GP-2021-K021854).

## Conflict of interest

The authors declare that the research was conducted in the absence of any commercial or financial relationships that could be construed as a potential conflict of interest.

## Publisher’s note

All claims expressed in this article are solely those of the authors and do not necessarily represent those of their affiliated organizations, or those of the publisher, the editors and the reviewers. Any product that may be evaluated in this article, or claim that may be made by its manufacturer, is not guaranteed or endorsed by the publisher.
